# Cytokine Profile in the Upper Airways of Patients With N‐ERD Obtained via a Minimally Invasive Method

**DOI:** 10.1155/jimr/2768458

**Published:** 2025-10-29

**Authors:** Karolina Frachowicz-Guerreiro, Adrian Gajewski, Rafał Ćwikliński, Marcin Kurowski, Maciej Chałubiński, Aleksandra Wardzyńska

**Affiliations:** ^1^ Department of Immunology and Allergy, Medical University of Lodz, Lodz, Poland, umed.pl

**Keywords:** allergic rhinitis, asthma, cytokines, drug allergy, N-ERD, nasal polyps, NSAIDs

## Abstract

**Introduction:**

NSAID‐exacerbated respiratory disease (N‐ERD) is a bronchial asthma phenotype characterized by the coexistence of NSAID hypersensitivity, lower airway symptoms, and severe chronic sinusitis with nasal polyps. This study examined the cytokine profiles in the upper airways of patients with N‐ERD compared to those of patients with NSAID‐tolerant asthma, allergic rhinitis (AR), and healthy controls.

**Methods:**

89 patients were included in the N‐ERD, AR, asthma, and control groups. The minimally invasive nasosorption technique was used to collect nasal mucosal lining fluid. Inflammatory mediators were quantified using a Bio‐Plex Multiplex System.

**Results:**

IL‐6 levels in nasal samples (NSs) were higher in all patient groups than in the controls. No significant differences were observed in the nasal levels of all analyzed cytokines between patients with N‐ERD and those with asthma or AR alone. Cluster analysis identified two distinct inflammatory phenotypes within the N‐ERD group based on nasal cytokine profiles, although these did not correlate with clinical features. A logistic regression model showed that only serum TNF‐α levels and the severity of nasal obstruction significantly differentiated patients with N‐ERD from the other groups.

**Conclusion:**

The results demonstrate that while patients with N‐ERD exhibit heterogeneity in upper airway inflammatory profiles, this does not necessarily translate into differences in clinical presentation.

**Summary**

• NSAIDs‐exacerbated respiratory disease (N‐ERD) is a heterogeneous disease.
• Upper airway inflammatory profiles of N‐ERD patients differ.
• Differences in clinical presentation do not necessarily correlate with upper airway inflammatory profile.

## 1. Introduction

NSAID‐exacerbated respiratory disease (N‐ERD) is one of the best‐defined phenotypes of bronchial asthma. The essence of this disease is chronic airway inflammation and the appearance of acute airway symptoms after exposure to strong cyclooxygenase‐1 (COX‐1) inhibitors. The pathogenesis of the syndrome is complex, involving many cells of both lymphoid and nonlymphoid origin as well as mediators secreted by them [1]. Most patients with N‐ERD are thought to predominantly have T‐2 inflammation with the presence of eosinophils. However, many studies have also shown an important role of non‐T2‐dependent cytokines, such as IFN‐γ or IL‐6, in this process [2]. Recently, there has been an increasing discussion about the diversity of the syndrome, indicating that it varies not only clinically but also in terms of inflammation. Studies using cluster analysis or data mining have shown the existence of subphenotypes differing in ventilation parameters, asthma control, and severity of upper airway symptoms [3]. It also appears that, in terms of inflammatory mediators found in the lower airways, subphenotypes of N‐ERD can be distinguished [4]. Recent suggestions indicate that cytokine levels measured in the upper respiratory tract might also fluctuate, further supporting the diversity within this group [5].

Among the proposed techniques for obtaining materials from the lower respiratory tract, sputum induction, bronchial lavages, and examination of tissues collected during bronchoscopy have been proposed. All these methods require interference (bronchoscopy) or have suboptimal reproducibility (induced sputum), are labor‐intensive, and cannot be used in a wide range of patients [6]. Some authors suggest that the evaluation of cytokines in the upper airways can be a satisfactory, although not ideal, surrogate for inflammation in the lower airways [7]. Methods for obtaining this material are simpler, more reproducible, and more patient‐friendly. Among the proposed techniques are nasal lavages and the use of swabs, sponges, and other devices coated with adsorptive materials. In the biological samples obtained in this way, it is possible to determine not only proteins but also nucleic acids or the presence of pathogens [8].

The aim of this pilot study was to compare the cytokine profiles collected using a minimally invasive method in the upper airways of patients with N‐ERD to those with other chronic airway conditions such as asthma and allergic rhinitis (AR). Mediator levels were also related to serum concentrations and clinical features.

## 2. Methods

### 2.1. Patients

In total 89 patients with N‐ERD (*n* = 13), AR (*n* = 22), asthma (*n* = 40), and healthy controls (*n* = 14) were recruited for the study. The patients were recruited in the outpatient clinic, had to meet the abovementioned inclusion criteria, and did not meet the exclusion criteria. All patients with N‐ERD and AA had concomitant AR. The control group was selected to meet similar demographic criteria (age, sex, and race) and did not have any disease in the study groups.

With regard to the recruitment to the study, as well as the methodology of the conducted research and the collection and processing of material, we used the methods previously described by us in the work by Gajewski et al. [9], which are briefly presented below.

The inclusion criteria were age 18–75 years, and a diagnosis of one or more of the following upper or lower respiratory diseases: AR (diagnosed according to ARIA criteria [10]), chronic rhinosinusitis with or without nasal polyps (diagnosed on the basis of EPOS [11]), bronchial asthma (based on GINA 2019 [12]), and N‐ERD (based on EAACI guidelines [1]). The control group consisted of subjects without any of the aforementioned diseases. The exclusion criteria were as follows: active atopic dermatitis, specific immunotherapy in the last 5 years, biological therapy within 6 months, aspirin therapy after desensitization (ATAD), active infectious diseases, exacerbation of chronic diseases, uncontrolled mental illnesses, malignant tumors in active status, taking immunosuppressive drugs, condition after organ transplantation, inability to communicate or understand the objectives of the study, or any active addictions.

Patients taking medications were asked to discontinue nasal steroids, montelukast, and antihistamines 7 days before the study and oral steroids 10 days before the visit. Inhaled steroids were discontinued 24 h before the visit.

All procedures were performed during a single visit. The patients completed a questionnaire about their symptoms, medications, and medical history. The ACQ‐6 (Asthma Control Questionnaire 6) was used to assess asthma control, the daily symptom score (DSS) to evaluate the severity of nasal symptoms, the mini‐Rhinoconjunctivitis Quality of Life Questionnaire (mRQLQ) to evaluate the quality of life of patients with AR, the SNOT‐22 (Sinonasal Outcome Test 22) to assess the severity of symptoms and quality of life in patients with CRS.

The study subjects performed a fractional exhaled nitric oxide (FeNO) measurement maneuver according to the ATS/ERS guidelines [13] using the HypAir FeNO (Medisoft, Belgium). The mean values of at least two measurements were analyzed. Spirometry was performed according to ERS standards [14] using a Vyntus system spirometer (Carefusion, San Diego, California, United States).

Atopy was evaluated using a panel of skin prick tests, including the following inhalant allergens: *Dermatophagoides pteronyssinus*, *Dermatophagoides farinae*, *Acarus siro*, *Tyrophagus putrescentiae*, *Lepidoglyphus destructor*, cat dander, *Alternaria tenuis*, birch pollen, a mix of grass pollen, mugwort pollen, nettle pollen, and ash pollen. The allergens selected were region‐specific. A positive result was defined as a wheal measuring 3 mm in diameter. Atopy was defined as the presence of at least one positive skin prick test result.

### 2.2. Blood Samples

Venous blood samples were collected in anticoagulant tubes (Sarstedt, Germany) to obtain serum and into EDTA tubes (Sarstedt, Germany) to determine differential blood counts.

Cell counts and differentiation were determined using an XN‐1000 Hematology Analyzer (Sysmex, Japan).

### 2.3. Nasal Sampling

Nasal mucosal lining fluid was collected using Nasosorption devices (Hunt Developments Ltd., United Kingdom). After short anterior rhinoscopy, the fluid was sampled by gently inserting the nasosorption into both nostrils for 60 s, while gently pressing on the wing of the nose. The procedure was repeated twice, after 60 min, with a 70 min interval in each nostril.

### 2.4. Sample Processing

The nasal samples (NSs) were eluted in 300 µL assay buffer (Ab‐33 k, Merck Millipore Life Science), centrifuged at 16,000 × *g* at 4°C for 20 min, and then pooled; the first nasosorption samples from both nostrils were pooled, and then the second and third from both nostrils were pooled. The samples were stored at −80°C.

Cytokine levels were quantified using a Bioplex Multiplex System (Bio‐Rad, München, Germany) using the Bio‐Plex Pro Human Cytokine 27‐plex Assay from Bio‐Rad according to the manufacturer’s instructions. A complete list of cytokines is provided in the supplement (Table S1). Only mediators that were present above the lower limit of normal in more than 40% of the samples for each material were included in the subsequent analysis (Table S1).

### 2.5. Statistical Analysis

Categorical variables were compared using Fisher’s exact test. Quantitative variables were presented as median and 25%–75% percentile and were compared using the Mann–Whitney *U* test or the Kruskal–Wallis test for multiple comparisons. Statistical analysis was performed using the Statistica software (StatSoft, Tulsa, OK, USA). Statistical significance was set at *p*  < 0.05.

A logistic regression model was developed using demographic information, clinical data, and mediator levels in both serum and nasal secretions to identify factors that differentiated patients with N‐ERD from other participants in the study. Cluster analysis using k‐means was performed to isolate inflammatory types among patients with N‐ERD. The levels of the mediators determined only during nasal discharge were included in the model. The clinical features of the clusters were compared by using the methods described above.

The study was approved by the local Bioethics Committee (Approval number: RNN/204/19/KE). All the study participants provided written informed consent.

## 3. Results

### 3.1. Group Characteristics

Patients with N‐ERD were significantly older than those with AR were. Patients with asthma had a lower FEV1%FVC (forced expiratory volume during 1 s to forced vital capacity expressed as a percentage) ratio than those in the control group. Patients with N‐ERD, asthma, and AR had significantly higher overall DSS scores, specifically DSS obstruction, itching, and watery discharge, than controls. Additionally, patients with asthma and subjects with AR had higher DSS sneezing and watery eyes than controls. Patients with AA and N‐ERD had more eosinophils in their peripheral blood than those with AR (Table 1).

**Table 1:** Comparison of demographic and clinical characteristics across study groups.

Features	Controls, *n* = 14	AR, *n* = 22	AA, *n* = 40	N‐ERD, *n* = 13	*p*	*p*	*p*	*p*	*p*	*p*
					AR vs controls	AA vs. AR	AR vs. N‐ERD	AA vs. controls	AA vs. N‐ERD	N‐ERD vs. controls
Age, years, Me (25%–75%)	32 (26–52)	33 (23–48)	41.5 (28.5–57)	49 (45–63)	ns	ns	0.01	ns	ns	ns
Sex, female, *n* (%)	8 (57.14%)	13 (59.09%)	13 (57.50%)	11 (84.62%)	ns	ns	ns	ns	ns	ns
BMI, Me (25%–75%)	22.62 (20.08–27.1)	22.09 (19.92–26.57)	25.6 (23.15–30)	26.03 (25.15–29.41)	ns	ns	ns	ns	ns	ns
Smoker, *n* (%)	2 (14.29%)	2 (9.09%)	3 (7.50%)	2 (15.38%)	ns	ns	ns	ns	ns	ns
FEV1%FVC, Me (25%–75%)	96 (93–101)	93 (93–100)	93 (84.5–93)	93 (91–96)	ns	ns	ns	0.047	ns	ns
FeNO [ppb], Me (25%–75%)	21 (16–21)	21 (18–21)	21 (17.5–24)	17 (16–21)	ns	ns	ns	ns	ns	ns
CRS, *n* (%)	na	na	17 (42.50%)	12 (92.31%)	ns	ns	ns	ns	ns	ns
Nasal polyps, *n* (%)	na	na	10 (25.00%)	8 (61.54%)	ns	ns	ns	ns	ns	ns
Perennial AR, *n* (%)	na	4 (18.18%)	30 (75.00%)	13 (100.00%)	ns	ns	ns	ns	ns	ns
Seasonal AR, *n* (%)	na	22 (100.00%)	35 (87.50%)	13 (100.00%)	ns	ns	ns	ns	ns	ns
DSS nasal obstruction, Me (25%–75%)	na	1 (0–2)	2 (1–2)	3 (2–3)	0.04	ns	Ns	<0.001	ns	<0.001
DSS itching, Me (25%–75%)	na	2 (0–2)	1 (0–3)	2 (0–2)	0.008	ns	Ns	0.002	ns	0.003
DSS sneezing, Me (25%–75%)	na	2 (1–3)	1.5 (1–2)	1 (0–2)	<0.001	ns	Ns	<0.001	ns	ns
DSS watery eyes, Me (25%–75%)	na	1 (0–2)	1 (0–2)	1 (0–2)	0.03	ns	Ns	0.007	ns	ns
DSS score, Me (25%–75%)	na	1.38 (0.75–1.75)	1.25 (0.75–2.25)	1.25 (0.75–2.25)	<0.001	ns	Ns	<0.001	ns	<0.001
mRQLQ score, Me (25%–75%)	na	2 (2–3)	2 (1–3)	3 (2–3)	ns	ns	ns	ns	ns	ns
GINA treatment step, Me (25%–75%)	na	na	3 (2.5–3)	2 (2–4)	ns	ns	ns	ns	ns	ns
ACQ6 score, Me (25%–75%)	na	na	1 (1–2)	1 (0–2)	ns	ns	ns	ns	ns	ns
Eos abs. [cells/uL], Me (25%–75%)	180 (70–180)	135 (90–170)	220 (150–365)	230 (180–520)	ns	0.04	0.01	ns	ns	ns
Asthma control (GINA)	—	—	—	—	—	—	—	—	—	—
Well controlled, *n* (%)	na	na	25 (64.10%)	7 (58.33%)	ns	ns	ns	ns	ns	ns
Partly controlled, *n* (%)	na	na	11 (28.21%)	3 (25.00%)	ns	ns	ns	ns	ns	ns
Uncontrolled, *n* (%)	na	na	3 (7.69%)	2 (16.67%)	ns	ns	ns	ns	ns	ns
Asthma severity (GINA)	—	—	—	—	—	—	—	—	—	—
Mild, *n* (%)	na	na	10 (26.32%)	4 (33.33%)	ns	ns	ns	ns	ns	ns
Moderate, *n* (%)	na	na	19 (50.00%)	7 (58.33%)	ns	ns	ns	ns	ns	ns
Severe, *n* (%)	na	na	9 (23.68%)	1 (8.33%)	ns	ns	ns	ns	ns	ns

*Note:* Eos, abs.—eosinophil absolute count, FEV1%FVC—forced expiratory volume during 1 s to forced vital capacity expressed as a percentage, N‐ERD—nonsteroidal anti‐inflammatory drugs exacerbated respiratory disease, ns—not statistically significant.

Abbreviations: AA, allergic asthma; ACQ6, Asthma Control Questionnaire 6; AR, allergic rhinitis; BMI, body mass index; CRS, chronic rhinosinusitis; DSS, daily symptom score; FeNO, fractional exhaled nitric oxide; GINA, Global Initiative for Asthma, Me, median; mRQLQ, mini‐Rhinoconjunctivitis Quality of Life Questionnaire; na, not applicablens.

### 3.2. Cytokine Levels Within the Groups

In NSs IL‐6 levels were significantly higher in each study group than in the control group. The levels of IFN‐γ, IL‐7, and IL‐13 were higher in NS patients with AR than in controls. TNF‐α levels were higher in the NS of patients with AR than in those with asthma and controls. In contrast, serum TNF‐α levels were higher in the N‐ERD group than in the control and AR groups (Figure 1A,B, Table S2).

Figure 1Notable variations in cytokine levels among study groups in (A) nasal and (B) serum samples. AR, allergic rhinitis; AA, allergic asthma; N‐ERD, nonsteroidal anti‐inflammatory drugs exacerbated respiratory disease; NS, nasal samples.

**Figure:**
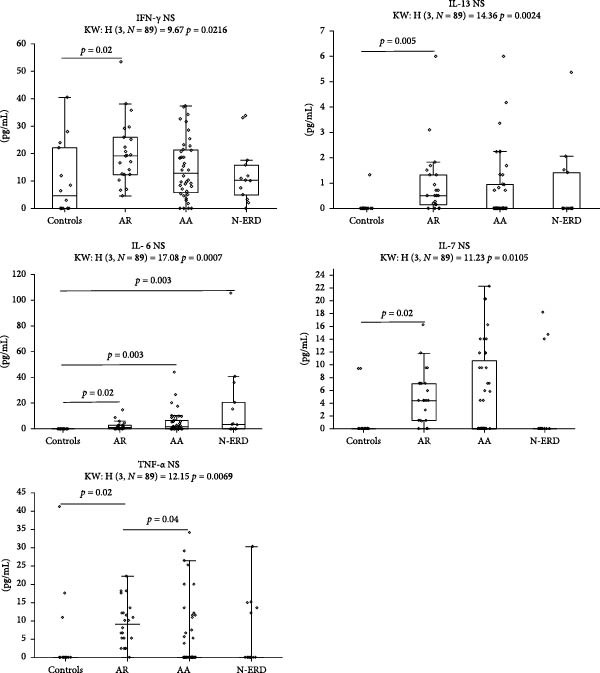
(A)

**Figure:**
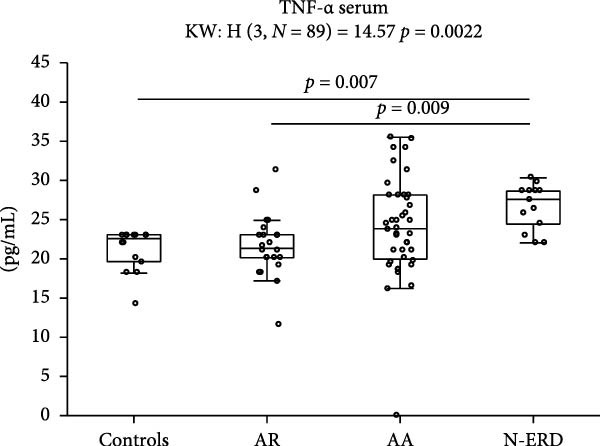
(B)

There was no correlation between nasal and serum mediator levels (Table S3).

### 3.3. Cluster Analysis Results

To investigate whether patients with N‐ERD had a specific cytokine profile in the upper airways, a cluster analysis was performed using the k‐means method. Only cytokine levels in the NSs were included in the model. The results showed the existence of three clusters (Figure 2A.). The 3rd cluster, represented mostly by patients with N‐ERD, included those with simultaneously high chemokine, T1 and T2 cytokine levels (Figure 2A,B). When we compared patients with N‐ERD from clusters 1 and 3, we found that they differed in the levels of mediators of NS (Table S4). However, these patients did not differ in clinical features or symptom severity (Table S5).

Figure 2(A) Normalized means of mediators in nasal samples in clusters determined by the k‐means method. (B) Graph of the cost sequence for the model.

**Figure:**
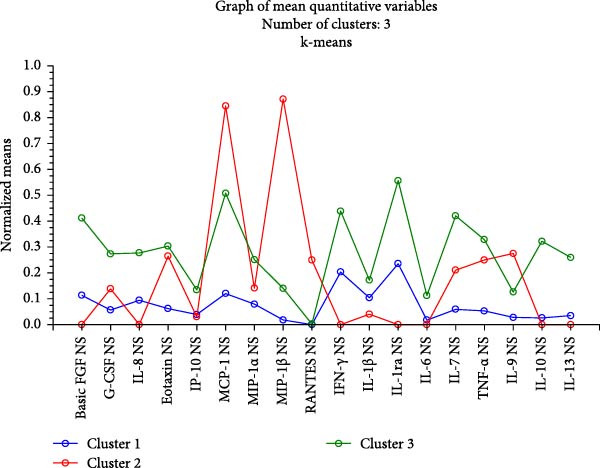
(A)

**Figure:**
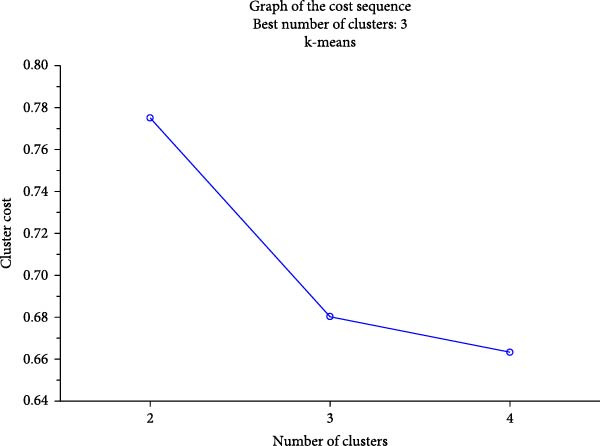
(B)

### 3.4. Results of the Linear Regression Model

Significant factors in the univariate analysis were included in the regression model. Of the factors included in the logistic regression model, only the severity of nasal blockage and serum TNF‐α levels were significant in distinguishing patients with N‐ERD (Table 2.).

**Table 2:** Results of the univariate and multivariate logistic regression.

Features	Univariate analysis		Multivariate analysis	
	OR (95 CI)	*p*	OR (95 CI)	*p*
Eos abs. (cells/uL)	1 (1–1.01)	0.0075	1 (1–1.01)	0.186
DSS nasal_obstruction	2.47 (1.3–4.71)	0.0058	2.66 (1.21–5.84)	0.015
DSS itching	1.17 (0.7–1.95)	0.558	—	—
DSS sneezing	0.8 (0.45–1.43)	0.4503	—	—
DSS watery eyes	1.15 (0.67–1.97)	0.6023	—	—
GINA asthma treatment step	0.67 (0.31–1.43)	0.2981	—	—
FEV1%FVC	1 (0.93–1.08)	0.9204	—	—
FENO [ppb]	0.92 (0.82–1.04)	0.1747	—	—
ACQ6 score	0.98 (0.42–2.31)	0.9693	—	—
mRQLQ score	1.26 (0.76–2.08)	0.3758	—	—
SNOT 22 score	1.03 (0.99–1.07)	0.2115	—	—
DSS score	1.46 (0.73–2.92)	0.2816	—	—
BMI	1.11 (0.99–1.24)	0.0821	—	—
Basic FGF NS	1.01 (0.95–1.08)	0.6766	—	—
Eotaxin NS	1.02 (0.97–1.06)	0.5005	—	—
G‐CSF NS	1 (1–1.01)	0.4275	—	—
IFN‐γ NS	0.98 (0.92–1.03)	0.3783	—	—
IL‐10 NS	1.3 (0.98–1.73)	0.0694	—	—
IL‐13 NS	1.07 (0.69–1.64)	0.7691	—	—
IL‐1β NS	1.06 (0.92–1.23)	0.4109	—	—
IL‐1ra NS	1 (1–1)	0.5377	—	—
IL‐6 NS	1.07 (1.01–1.13)	0.0178	1.04 (0.96–1.12)	0.327
IL‐7 NS	0.97 (0.87–1.08)	0.5848	—	—
IL‐8 NS	1 (1–1)	0.0189	1 (1–1)	0.165
IL‐9 NS	1 (0.98–1.02)	0.9274	—	—
IP‐10 NS	1 (1–1)	0.4082	—	—
MCP‐1 NS	1.01 (0.96–1.06)	0.7931	—	—
MIP‐1 α NS	1.22 (1–1.49)	0.0555	—	—
MIP‐1β NS	1 (0.99–1.01)	0.8071	—	—
RANTES NS	1 (0.99–1.01)	0.8547	—	—
TNF‐α NS	1 (0.94–1.07)	0.9451	—	—
Basic FGF Serum	0.99 (0.93–1.04)	0.5779	—	—
Eotaxin Serum	0.99 (0.96–1.01)	0.2574	—	—
G‐CSF Serum	0.99 (0.98–1.01)	0.2437	—	—
IL‐4 Serum	0.93 (0.51–1.7)	0.8215	—	—
IL‐8 Serum	1.07 (0.89–1.28)	0.504	—	—
IL‐9 Serum	1 (0.98–1.02)	0.9553	—	—
IP‐10 Serum	1 (0.99–1)	0.2676	—	—
MCP‐1 Serum	0.98 (0.93–1.03)	0.3166	—	—
MIP‐1 α Serum	0.81 (0.41–1.63)	0.5581	—	—
MIP‐1β Serum	1.01 (0.98–1.05)	0.5513	—	—
PDGF‐BB Serum	1 (1–1)	0.217	—	—
RANTES Serum	1 (1–1)	0.2523	—	—
TNF‐α Serum	1.17 (1.03–1.33)	0.0161	1.19 (1.01–1.39)	0.033

*Note:* Eos abs.—absolute eosinophil count, FEV1%FVC—forced expiratory volume in 1 s (FEV1) to forced vital capacity (FVC), expressed as a percentage.

Abbreviations: ACQ‐6, Asthma Control Questionnaire 6; BMI, body mass index; DSS, daily symptom score; FeNO, fractional exhaled nitric oxide; FGF, fibroblast growth factor; G‐CSF, granulocyte‐colony stimulating factor; GINA, global initiative asthma; IFN‐y, interferon gamma; IL, interleukin; MCP, monocyte chemoattractant protein; MIP, macrophage inflammatory proteins; mRQLQ, mini‐Rhinoconjunctivitis Quality of Life Questionnaire, NS, nadal samples; PDGF, platelet‐derived growth factor; RANTES, regulated upon activation of normal T‐cell expressed and secreted, SNOT –22, sinonasal outcome test 2, TNF, tumor necrosis factor.

## 4. Discussion

In this study, we found no differences in the levels of cytokines in NSs from patients with N‐ERD compared to those with asthma or AR alone. This might be attributed to the fact that we tried to balance the groups and deliberately selected patients with N‐ERD who simultaneously had comorbid AR. Also in the group of asthmatics, all subjects were diagnosed with seasonal or perennial AR. All these entities are classified as diseases with predominant eosinophilic inflammation in the airways, with a leading role for T2‐dependent inflammatory cytokines. Interestingly, the only cytokine that was significantly different in all groups compared with the control group was IL‐6, a pleiotropic cytokine that is part of the acute phase response. IL‐6 is an important element in the pathogenesis of AR [15] and asthma. It is produced by mast cells, fibroblasts, and epithelial cells, and its presence may be important for the differentiation of Th2 lymphocytes from naive Th cells [16]. In patients with asthma, IL‐6 levels are associated with ventilation parameters [17] and disease control [18]. Studies on IL‐6 have also encompassed individuals with N‐ERD [5].

Observations made during treatment with dupilumab, a monoclonal antibody that blocks the action of IL‐4 and IL‐13, indicate that this drug significantly reduces the levels of IL‐6 in the nasal fluid of patients with N‐ERD [19]. The reduction in cytokine levels was likely due to the effect of dupilumab on various cell types, including mast cells and monocytes, and probably on nasal fibroblasts. The authors of this paper speculate that the reduction in IL‐6 may be partially responsible for the therapeutic benefits of dupilumab, as IL‐6 is known to have various downstream effects, including the induction of respiratory epithelial cell disruption. In a study by Pezato et al. [20] the baseline serum concentration of IL‐6 was a marker of systemic response during the aspirin challenge test. The authors hypothesized that alveolar macrophages, nasal epithelial cells, and fibroblasts may be potential sources of this cytokine. Moreover, its production may be associated with the increased production of cysteinyl leukotrienes, which may then activate various cells to produce IL‐6. In the study cited above, IL‐8 was another factor correlated with increased sensitivity to aspirin. The level of this chemokine was also higher at baseline than in those without N‐ERD; however, it did not change during the ASA challenge. However, patients with higher baseline IL‐8 levels responded to lower ASA doses. The authors also observed a correlation between IL‐8 and IL‐5R, supporting the hypothesis that IL‐8 is also a chemoattractant for eosinophils. In our study, IL‐8 levels in NS were also a differentiating factor between patients with N‐ERD and the other groups in a univariate analysis of the linear regression model; however, this observation was not confirmed by multivariate analysis.

Of the factors identified as differentiating patients with N‐ERD from other study participants, only the severity of nasal obstruction and serum TNF‐α levels were entered into the model. TNF‐α is a pro‐inflammatory cytokine with pleiotropic effects and is secreted by immune cells, fibroblasts, and epithelium [21]. The potential role of TNF‐α in asthma is related to its chemotactic properties in inflammatory cells and stimulation of the proliferation of muscle cells or fibroblasts, which may be important in processes related to remodeling in asthma [22]. Polymorphisms in the TNF‐α gene have been described as potentially associated with an increased risk of N‐ERD [23, 24, 25].

By examining only the mediator levels in NSs, we identified two distinct inflammatory phenotypes within our N‐ERD group (Table 3). More than half of the patients with N‐ERD represent a subtype in which both growth factors and chemokines, as well as T1‐ and T2‐dependent cytokines, are significantly elevated, with no predominance of a particular group of mediators. Importantly, among patients with N‐ERD, the two groups did not differ significantly in terms of symptoms or asthma severity, despite their different airway inflammatory signatures. A similar approach to analyzing the results was taken earlier by Scott et al. [5]. He identified three distinct inflammatory clusters in patients with N‐ERD based on cytokine signatures determined in nasal mucosa samples: low inflammatory burden cluster, type 2‐high cluster, and type 2‐low but type 1/type 3‐high cluster. Interestingly, the cytokine elevated in the second cluster was also IL‐6. Despite some observed differences in the clinical features within the groups, these differences were not statistically significant.

**Table 3:** Affiliation of each group of study subjects to clusters determined by k‐means method.

Clusters	Controls, *n* = 14	AR, *n* = 22	AA, *n* = 40	N‐ERD, *n* = 13
Cluster 1, *n* = 52	8 (57.14%)	13 (59.09%)	25 (62.50%)	6 (46.15%)
Cluster 2, *n* = 4	4 (28.57%)	0 (0.00%)	0 (0.00%)	0 (0.00%)
Cluster 3, *n* = 33	2 (14.29%)	9 (40.91%)	15 (37.50%)	7 (53.85%)

*Note:* N‐ERD, nonsteroidal anti‐inflammatory drug exacerbated respiratory disease.

Abbreviations: AA, allergic asthma; AR, allergic rhinitis.

Our study had certain limitations. First, a potential source of bias is the small and unrepresentative sample size. Second, the groups did not constitute a pure model, as there were predominantly coexisting and potentially overlapping disease entities. Furthermore, given the exploratory nature of our study, we used a commercially available panel encompassing a broad range of cytokines, growth factors, and chemokines. Consequently, some mediators were not detectable in our samples, necessitating the use of more sensitive methods in such instances. We are also aware that the method of collecting biological material using absorbent material allows for the assessment of only part of the inflammatory process occurring in the airways. This method makes it possible to evaluate soluble proteins on the surface of epithelium; however, it does not provide insight into the deeper parts of the airways, like cellular composition and the processes taking place in the deeper layers.

## 5. Conclusions

Nevertheless, we have shown that material collected using a minimally invasive method is a good source of mediators in patients with N‐ERD and allows us to distinguish different inflammatory types within this group. In order to thoroughly assess the potential of this approach for diagnostic or prognostic use in patients with N‐ERD, further investigation is crucial. It is vital to explore how effective this method is in evaluating other biomarkers that play a significant role in the disease’s pathogenesis. An especially interesting research focus would be its capability in assessing the production of lipid mediators, which are essential to the disease’s development.

## Ethics Statement

This study was approved by the Ethics Committee of the Medical University of Lodz (Approval number RNN/204/19/KE). All procedures involving human participants were performed in accordance with the Ethical Standards of the Institutional and National Research Committee and the 1964 Helsinki Declaration and its later amendments. Written informed consent was obtained from all the participants included in the study.

## Conflicts of Interest

The authors declare no conflicts of interest.

## Author Contributions

**Karolina Frachowicz-Guerreiro:** investigation, data curation, writing – original draft, visualization. **Adrian Gajewski and Rafał Ćwikliński:** investigation. **Marcin Kurowski:** methodology, investigation, writing – review and editing, supervision. **Maciej Chałubiński:** resources, writing – review and editing, project administration, funding acquisition. **Aleksandra Wardzyńska:** conceptualization, methodology, formal analysis, investigation, writing – original draft, visualization, supervision.

## Funding

This study was supported by the EIT Health (ADAPT—Airway Diseases, Analysis, and Prevention; Grant 19065), a body of the European Union.

## Supporting Information

Additional supporting information can be found online in the Supporting Information section.

## Supporting information


**Supporting Information** Table S1. Percentage (%) of results above lower limit of normal (LLN) in nasal and serum samples. Table S2. Comparison of mediator levels between study groups. Table S3. Correlation between mediator levels in serum and nasal samples in whole study group. Table S4. Comparison of mediator levels between two groups of N‐ERD patients identified through k‐means analysis. Table S5. Comparison of clinical characteristics between two groups of N‐ERD patients identified through k‐means analysis.

## Data Availability

The data that support the findings of this study are available from the corresponding author upon reasonable request.

## References

[1] Kowalski M. L. , Agache I. , and Bavbek S. , et al.Diagnosis and Management of NSAID-Exacerbated Respiratory Disease (N-ERD)-a EAACI Position Paper, Allergy. (2019) 74, no. 1, 28–39, 10.1111/all.13599, 2-s2.0-85054312778. 30216468

[2] Borish L. , Hise K. , Negri J. , Caughey R. , and Steinke J. W. , Generation of the Aspirin-Exacerbated Respiratory Disease (AERD) Phenotype by Th1/Th2 Cytokines, Journal of Allergy and Clinical Immunology. (2005) 115, no. 2, 10.1016/j.jaci.2004.12.478, S116.

[3] Bochenek G. , Kuschill-Dziurda J. , Szafraniec K. , Plutecka H. , Szczeklik A. , and Nizankowska-Mogilnicka E. , Certain Subphenotypes of Aspirin-Exacerbated Respiratory Disease Distinguished by Latent Class Analysis, Journal of Allergy and Clinical Immunology. (2014) 133, no. 1, 98–103.e6, 10.1016/j.jaci.2013.07.004, 2-s2.0-84891738600. 23993879

[4] Jakiela B. , Soja J. , and Sladek K. , et al.Heterogeneity of Lower Airway Inflammation in Patients With NSAID-Exacerbated Respiratory Disease, Journal of Allergy and Clinical Immunology. (2021) 147, no. 4, 1269–1280, 10.1016/j.jaci.2020.08.007. 32810516

[5] Scott W. C. , Cahill K. N. , and Milne G. L. , et al.Inflammatory Heterogeneity in Aspirin-Exacerbated Respiratory Disease, Journal of Allergy and Clinical Immunology. (2021) 147, no. 4, 1318–1328.e5, 10.1016/j.jaci.2020.11.001. 33189729 PMC8035132

[6] Baumann R. , Untersmayr E. , and Zissler U. M. , et al.Noninvasive and Minimally Invasive Techniques for the Diagnosis and Management of Allergic Diseases, Allergy. (2021) 76, no. 4, 1010–1023, 10.1111/all.14645. 33128851

[7] Zissler U. M. , Ulrich M. , and Jakwerth C. A. , et al.Biomatrix for Upper and Lower Airway Biomarkers in Patients With Allergic Asthma, Journal of Allergy and Clinical Immunology. (2018) 142, no. 6, 1980–1983, 10.1016/j.jaci.2018.07.027, 2-s2.0-85053177188. 30121290

[8] Hansel T. T. , Tunstall T. , and Trujillo-Torralbo M. B. , et al.A Comprehensive Evaluation of Nasal and Bronchial Cytokines and Chemokines Following Experimental Rhinovirus Infection in Allergic Asthma: Increased Interferons (IFN-γ and IFN-λ) and Type 2 Inflammation (IL-5 and IL-13), EBioMedicine. (2017) 19, 128–138, 10.1016/j.ebiom.2017.03.033, 2-s2.0-85016488815. 28373098 PMC5440599

[9] Gajewski A. , Bekier A. , and Frachowicz-Guereirro K. , et al.Analysis of miRNA Expression in Patients With NSAID-Exacerbated Respiratory Disease, Allergy, Asthma & Immunology Research. (2025) 17, no. 2, 226–240, 10.4168/aair.2025.17.2.226. PMC11982641 40204507

[10] Bousquet J. , Schünemann H. J. , and Togias A. , et al.Next-generation Allergic Rhinitis and Its Impact on Asthma (ARIA) Guidelines for Allergic Rhinitis Based on Grading of Recommendations Assessment, Development and Evaluation (GRADE) and Real-World Evidence, Journal of Allergy and Clinical Immunology. (2020) 145, no. 1, 70–80.e3, 10.1016/j.jaci.2019.06.049, 2-s2.0-85073509724. 31627910

[11] Fokkens W. J. , Lund V. J. , and Hopkins C. , et al.European Position Paper on Rhinosinusitis and Nasal Polyps 2020, Rhinology journal. (2020) 58, no. Suppl S29, 1–464, 10.4193/Rhin20.600. 32077450

[12] Global Strategy for Asthma Management and Prevention , Global Initiative for Asthma, 2019, https://www.ginasthma.org.

[13] American Thoracic Society and European Respiratory Society , ATS/ERS Recommendations for Standardized Procedures for the Online and Offline Measurement of Exhaled Lower Respiratory Nitric Oxide and Nasal Nitric Oxide, 2005, American Journal of Respiratory and Critical Care Medicine. (2005) 171, no. 8, 912–930, 10.1164/rccm.200406-710ST, 2-s2.0-17644363081. 15817806

[14] Miller M. R. , Hankinson J. , and Brusasco V. , et al.Standardisation of Spirometry, European Respiratory Journal. (2005) 26, no. 2, 319–338, 10.1183/09031936.05.00034805, 2-s2.0-21744460289. 16055882

[15] Gao S. , Yu L. , and Zhang J. , et al.Expression and Clinical Significance of VCAM-1, IL-6, and IL-17A in Patients With Allergic Rhinitis, Annals of Palliative Medicine. (2021) 10, no. 4, 4516–4522, 10.21037/apm-21-546. 33966399

[16] Diehl S. and Rincón M. , The Two Faces of IL-6 on Th1/Th2 Differentiation, Molecular Immunology. (2002) 39, no. 9, 531–536, 10.1016/S0161-5890(02)00210-9, 2-s2.0-0036889996. 12431386

[17] Neveu W. A. , Allard J. L. , and Raymond D. M. , et al.Elevation of IL-6 in the Allergic Asthmatic Airway Is Independent of Inflammation but Associates With Loss of Central Airway Function, Respiratory Research. (2010) 11, no. 1, 10.1186/1465-9921-11-28, 2-s2.0-77952536377, 28. 20205953 PMC2842243

[18] Tillie-Leblond I. , Pugin J. , and Marquette C. H. , et al.Balance Between Proinflammatory Cytokines and Their Inhibitors in Bronchial Lavage From Patients With Status Asthmaticus, American Journal of Respiratory and Critical Care Medicine. (1999) 159, no. 2, 487–494, 10.1164/ajrccm.159.2.9805115, 2-s2.0-0008249094. 9927362

[19] Schneider S. , Poglitsch K. , and Morgenstern C. , et al.Dupilumab Increases Aspirin Tolerance in NSAID-Exacerbated Respiratory Disease, European Respiratory Journal. (2023) 61, no. 3, 10.1183/13993003.01335-2022, 2201335. 36549708 PMC10017890

[20] Pezato R. , Świerczyńska-Krępa M. , and Niżankowska-Mogilnicka E. , et al.Systemic Expression of Inflammatory Mediators in Patients With Chronic Rhinosinusitis and Nasal Polyps With and Without Aspirin Exacerbated Respiratory Disease, Cytokine. (2016) 77, 157–167, 10.1016/j.cyto.2015.10.011, 2-s2.0-84947794472. 26615369

[21] Berry M. , Brightling C. , Pavord I. , and Wardlaw A. , TNF-α in Asthma, Current Opinion in Pharmacology. (2007) 7, no. 3, 279–282, 10.1016/j.coph.2007.03.001, 2-s2.0-34248374283. 17475560

[22] Brightling C. , Berry M. , and Amrani Y. , Targeting TNF-Alpha: A Novel Therapeutic Approach for Asthma, Journal of Allergy and Clinical Immunology. (2008) 121, no. 1, 5–10, 10.1016/j.jaci.2007.10.028, 2-s2.0-38149091442. 18036647 PMC3992375

[23] Kim S. H. , Ye Y. M. , and Lee S. K. , et al.Association of TNF-Alpha Genetic Polymorphism With HLA DPB1 ^∗^0301, Clinical & Experimental Allergy. (2006) 36, no. 10, 1247–1253, 10.1111/j.1365-2222.2006.02567.x, 2-s2.0-33749326016. 17014432

[24] Pavón-Romero G. F. , Reséndiz-Hernández J. M. , and Ramírez-Jiménez F. , et al.Single Nucleotide Polymorphisms in TNF are Associated With Susceptibility to Aspirin-Exacerbated Respiratory Disease but Not to Cytokine Levels: A Study in Mexican Mestizo Population, Biomarkers in Medicine. (2017) 11, no. 12, 1047–1055, 10.2217/bmm-2017-0164, 2-s2.0-85038374302. 29172674

[25] Reigada-Rivera M. L. , Lozano C. S. , and Rodilla E. M. , et al.Polymorphisms in Human *IL4*, *IL10*, and, *TNF*, Genes are Associated With an Increased Risk of Developing NSAID-Exacerbated Respiratory Disease, Genes. (2022) 13, no. 4, 10.3390/genes13040605, 605. 35456412 PMC9031626

