# Supplementary material for: Cytokine Profile in the Upper Airways of Patients With N‐ERD Obtained via a Minimally Invasive Method
Source: J Immunol Res. 2025 Oct 29;2025:2768458. doi: 10.1155/jimr/2768458 (PMC12572634; doi:10.1155/jimr/2768458)
Supplement: Supplementary file 1 — Supporting Information Table S1. Percentage (%) of results above lower limit of normal (LLN) in nasal and serum samples. Table S2. Comparison of mediator levels between study groups. Table S3. Correlation between mediator levels in serum and nasal samples in whole study group. Table S4. Comparison of mediator levels between two groups of N‐ERD patients identified through k‐means analysis. Table S5. Comparison of clinical characteristics between two groups of N‐ERD patients identified through k‐means analysis. [file JIMR-2025-2768458-s001.pdf]

Table 1s. Percentage (%) of results above lower limit of normal (LLN) in nasal and serum samples.

Nasal samples, n=89		Serum samples, n=89	
**Mediator**	**%**	**Mediator**	**%**
**Basic FGF**	49.4	**Basic FGF**	48.3
**Eotaxin**	92.1	**Eotaxin**	92.1
**G-CSF**	73.0	**G-CSF**	89.9
**GM-CSF**	9.0	**GM-CSF**	3.4
**IFN-γ**	87.6	**IFN-γ**	32.6
**IL-10**	41.6	**IL-10**	4.5
**IL-12**	22.5	**IL-12**	6.7
**IL-13**	44.9	**IL-13**	19.1
**IL-15**	5.6	**IL-15**	0.0
**IL-17**	32.6	**IL-17**	11.2
**IL-1β**	88.8	**IL-1 β**	23.6
**IL-1ra**	94.4	**IL-1ra**	28.1
**IL-2**	2.2	**IL-2**	2.2
**IL-4**	14.6	**IL-4**	84.3
**IL-5**	32.6	**IL-5**	3.4
**IL-6**	53.9	**IL-6**	5.6
**IL-7**	48.3	**IL-7**	36.0
**IL-8**	100.0	**IL-8**	68.5
**IL-9**	67.4	**IL-9**	92.1
**IP-10**	98.9	**IP-10**	92.1
**MCP-1**	78.7	**MCP-1**	92.1
**MIP-1 α**	85.4	**MIP-1α**	79.8
**MIP-1β**	68.5	**MIP-1 β**	92.1
**PDGF-BB**	20.2	**PDGF-BB**	92.1
**RANTES**	48.3	**RANTES**	92.1
**TNF-α**	55.1	**TNF- α**	91.0

**VEGF**	3.4	**VEGF**	6.7

Table 2S. Comparison of mediator levels between study groups.

	Controls, n=14	AR, n=22	AA, n=23	N-ERD, n=13	p<br>AR vs controls	p<br>AA vs. AR	p<br>AR vs N-ERD	p<br>AA vs controls	p<br>AA vs N-ERD	p<br>N-ERD vs controls
**Nasal samples**										
**Basic FGF**	0 (0-15.55)	10.11 (0-17.08)	0 (0-9.03)	8.6 (0-17.08)	ns	ns	ns	ns	ns	ns
**Eotaxin**	3.5 (0.45-4.96)	7.98 (4.07-11.63)	4.1 (1.25-7.64)	4.26 (1.81-13.96)	ns	ns	ns	ns	ns	ns
**G-CSF**	45.03 (15.42-90.54)	26.72 (7.01-135.64)	74.03 (0-150.49)	48.2 (0-153.77)	ns	ns	ns	ns	ns	ns
**IFN-γ**	4.67 (0-22.13)	19.19 (12.31-25.94)	12.89 (5.79-21.32)	10.26 (4.95-15.79)	0.02	ns	ns	ns	ns	ns
**IL-10**	0 (0-0)	0 (0-1.08)	0 (0-2.96)	0 (0-3.59)	ns	ns	ns	ns	ns	ns
**IL-13**	0 (0-0)	0.5 (0.15-1.32)	0 (0-0.95)	0 (0-1.41)	0.005	ns	ns	ns	ns	ns
**IL-1β**	1.32 (0-3.92)	2.09 (1.68-3.55)	2.23 (1.25-3.7)	2.71 (1.97-3.45)	Ns	ns	ns	ns	ns	ns
**IL-1ra**	3169.1 (0-10023.22)	8037.59 (5544.2-11726)	7820.42 (3898.18-12591.82)	7621.05 (4570.45-12695.24)	Ns	ns	ns	ns	ns	ns
**IL-6**	0 (0-0)	1.14 (0-2.8)	1.64 (0-6.5)	3.47 (0-20.58)	0.02	ns	ns	0.03	ns	0.003
**IL-7**	0 (0-0)	4.4 (1.31-7.04)	0 (0-10.63)	0 (0-0)	0.02	ns	ns	ns	ns	ns
**IL-8**	71.02 (5.9-296.84)	217.55 (116.12-369.7)	194.56 (105.98-345.56)	276.79 (171.49-540.78)	Ns	ns	ns	ns	ns	ns
**IL-9**	7.11 (0-8.96)	9.46 (0-17.5)	5.11 (0-17.28)	18.39 (3.18-24.5)	Ns	ns	ns	ns	ns	ns
**IP-10**	326.78 (237.2-454.66)	324.11 (176.18-1168)	528.61 (244.02-1251.83)	187.19 (153.52-473.33)	Ns	ns	ns	ns	ns	ns
**MCP-1**	9.36 (0-28.72)	10.53 (5.91-14.81)	8.11 (0-13.32)	8.79 (0-20.5)	Ns	ns	ns	ns	ns	ns

**MIP-1α**	1 (0-1.43)	0.73 (0.23-1.61)	1.01 (0.54-2)	2.06 (0.76-3.41)	Ns	ns	ns	ns	ns	ns
**MIP-1β**	7.31 (0-134.27)	8.08 (1.83-15.83)	4.64 (0-15.13)	10.16 (0-47.85)	Ns	ns	ns	ns	ns	ns
**RANTES**	0 (0-0)	3.49 (0-12.59)	0 (0-14.15)	0 (0-12.63)	Ns	ns	ns	ns	ns	ns
**TNF-α**	0 (0-0)	9.13 (5.3-12.18)	0 (0-11.18)	0 (0-13.52)	0.02	0.04	ns	ns	ns	ns
**Serum**										
**Basic FGF**	0 (0-0)	3.3 (0-16.63)	10.8 (0-20.06)	0 (0-18.98)	Ns	ns	ns	ns	ns	ns
**Eotaxin**	63.11 (33.73-63.11)	63.16 (52.88-74.71)	64.25 (40.92-86.98)	46.37 (37.61-66.57)	Ns	ns	ns	ns	ns	ns
**G-CSF**	82.31 (38.22-82.31)	94.26 (64.52-123.4)	84.42 (52.95-135.08)	88.65 (40.75-102.97)	ns	ns	ns	ns	ns	ns
**IL-4**	1.95 (0.79-1.98)	1.92 (1.68-2.33)	2.26 (1.71-2.59)	1.85 (1.57-2.04)	ns	ns	ns	ns	ns	ns
**IL-8**	3.64 (0-3.64)	3.64 (2.44-4.46)	3.64 (0-4.89)	4.46 (0-6.13)	ns	ns	ns	ns	ns	ns
**IL-9**	296.68 (295.6-296.68)	296.68 (288.6-308.53)	298.3 (283.75-324.68)	299.37 (285.9-312.84)	ns	ns	ns	ns	ns	ns
**IP-10**	327.09 (327.09-327.73)	327.02 (267.74-420.16)	328.06 (252.79-529.05)	327.09 (187.95-431.35)	ns	ns	ns	ns	ns	ns
**MCP-1**	21.31 (9.96-21.31)	21.6 (18.2-28.24)	23 (12.99-37.2)	20.9 (14.01-26.9)	ns	ns	ns	ns	ns	ns
**MIP-1α**	1.24 (1.24-1.35)	1.52 (0.83-2.04)	1.18 (0.57-1.96)	1.24 (0.83-1.57)	ns	ns	ns	ns	ns	ns
**MIP-1β**	183.96 (174.06-183.96)	182.78 (173.29-191.95)	182.08 (167.91-191.57)	184.11 (177.12-190.86)	ns	ns	ns	ns	ns	ns
**PDGF-BB**	1715.37 (972.8-1715.37)	1678.63 (1455.55-2446.09)	1803.15 (1289.03-2515.56)	1365.7 (1081.94-2091.28)	ns	ns	ns	ns	ns	ns

**RANTES**	14532.82 (13708.68-14532.82)	14486.01 (13138.3-16123.24)	15246.67 (14106.91-16721.83)	13953.15 (13417.27-14825.31)	ns	ns	ns	ns	ns	ns
**TNF-α**	22.57 (19.65-23.08)	21.34 (20.13-23.08)	23.84 (19.97-28.14)	27.58 (24.44-28.65)	ns	ns	0.009	ns	ns	0.007

Table 3s. Correlation between mediator levels in serum and nasal samples in whole study group.

	Basic FGF NS	Eotaxin NS	G-CSF NS	IL-8 NS	IL-9 NS	IP-10 NS	MCP-1 NS	MIP-1alfa NS	MIP-1beta NS	RANTES NS	TNF-α NS
**Basic FGF Serum**	-0.12	-0.26	-0.20	-0.09	-0.19	-0.26	-0.18	-0.28	-0.35	-0.18	-0.07
**Eotaxin Serum**	-0.22	-0.09	-0.23	-0.06	-0.11	-0.09	-0.12	-0.12	-0.17	-0.12	-0.14
**G-CSF Serum**	-0.12	-0.02	-0.09	-0.02	0.00	0.01	-0.09	-0.02	-0.03	0.01	-0.05
**IL-8 Serum**	-0.07	0.03	0.02	0.01	0.04	-0.10	-0.06	-0.09	0.00	0.02	-0.03
**IL-9 Serum**	0.12	-0.01	-0.04	0.03	0.08	0.05	0.04	0.05	0.01	0.00	0.13
**IP-10 Serum**	-0.22	-0.09	-0.08	-0.07	-0.11	-0.11	-0.10	0.05	-0.02	-0.21	-0.18
**MCP-1 Serum**	-0.16	-0.14	-0.23	-0.12	-0.12	-0.15	-0.18	-0.21	-0.19	-0.07	-0.22
**MIP-1alfa Serum**	-0.03	0.00	0.11	0.04	0.06	0.06	-0.02	0.05	0.10	0.13	-0.03
**MIP-1beta Serum**	0.03	0.02	0.09	-0.03	0.18	-0.06	-0.01	0.01	0.09	-0.01	0.16
**RANTES Serum**	0.00	0.04	0.09	0.02	0.15	0.13	0.07	0.12	0.04	0.09	0.11
**TNF-α Serum**	0.10	0.06	0.07	0.08	0.30	0.09	0.07	0.13	0.14	0.11	0.10

The table shows the R values of the Sperman correlation coefficient. Those with $p<0.05$ are marked in red.

Table 4s. Comparison of mediator levels between two groups of N-ERD patients identified through k-means analysis.

	N-ERD, cluster 1, n=6	N-ERD, cluster 3, n=7	p
**Nasal samples**			
**Basic FGF**	0 (0-8,6)	17,08 (0-19,59)	ns
**Eotaxin**	1,56 (0,34-1,96)	13,96 (7,2-24,79)	0,002
**G-CSF**	0 (0-21,92)	153,77 (48,2-528,88)	0,005
**IFN-γ**	4,13 (2,11-7,47)	15,79 (10,26-33,05)	0,005
**IL-10**	0 (0-0)	3,59 (0-6,31)	0,035
**IL-13**	0 (0-0)	1,41 (0-2,06)	ns
**IL-1β**	2,34 (0,74-3,45)	2,71 (2,16-9,87)	ns
**IL-1ra**	4164,15 (2330,14-6852,08)	12695,24 (8448,33-20344,56)	0,0047
**IL-6**	0 (0-0)	20,58 (4-40,81)	0,0012
**IL-7**	0 (0-0)	0 (0-14,74)	ns
**IL-8**	155,11 (64,7-251,15)	540,78 (276,79-1427,22)	0,014
**IL-9**	1,59 (0-3,6)	24,5 (18,39-38,59)	0,005
**IP-10**	129,44 (61,38-183,99)	473,33 (385,5-818,46)	0,008
**MCP-1**	0 (0-3,04)	20,5 (8,79-29,56)	0,008
**MIP-1α**	0,73 (0,69-0,83)	3,41 (2,06-9,56)	0,008
**MIP-1β**	0 (0-1,54)	47,85 (21,15-59,93)	0,002
**RANTES**	0 (0-0)	12,63 (6,65-26,22)	0,008
**TNF-α**	0 (0-0)	13,52 (0-15,04)	0,035
**Serum**			
**Basic FGF**	17,81 (0-21,13)	0 (0-0)	ns
**Eotaxin**	52,07 (42,45-77,92)	41,08 (26,14-66,57)	ns
**G-CSF**	66,41 (40,75-101,24)	92,86 (30,84-104,03)	ns
**IL-4**	1,89 (1,57-2,71)	1,85 (0,79-2,04)	ns
**IL-8**	3,75 (0-4,46)	6,13 (0-7,15)	ns
**IL-9**	292,64 (279,89-313,37)	299,91 (289,16-312,84)	ns
**IP-10**	342,49 (305,16-445,6)	234,16 (182,25-431,35)	ns
**MCP-1**	21,34 (14,01-26,9)	20,41 (11,96-27,8)	ns
**MIP-1α**	1,24 (0,83-1,46)	1,24 (0,65-1,96)	ns
**MIP-1β**	176,41 (175,67-181,71)	190,86 (184,11-195,51)	0,014
**PDGF-BB**	1629,03 (1346,98-2125,3)	1147,53 (905,54-2091,28)	ns
**RANTES**	13543,32 (12124,47-14713,34)	14743,52 (13898,14-15253,61)	ns
**TNF-α**	25,15 (23,01-26,32)	28,65 (27,58-28,69)	ns

Table 5s. Comparison of clinical characteristics between two groups of N-ERD patients identified through k-means analysis.

	N-ERD, cluster 1, n=6	N-ERD, cluster 3, n=7	p
**age, years, Me (25-75%)**	47 (44-61)	50 (48-67)	ns
**sex, female, n (%)**	5 (83.33%)	6 (85.71%)	ns
**BMI, Me (25-75%)**	25.63 (23.15-26.13)	29.34 (25.15-35.94)	ns
**Smoker, n (%)**	0 (0%)	2 (28.57%)	ns
**FEV1%FVC, Me (25-75%)**	94.5 (91-97)	93 (88-93)	ns
**FeNO [ppb], Me (25-75%)**	16.5 (14-17)	21 (17-25)	ns
**SNOT22, score, Me (25-75%)**	41.5 (30-60)	43 (24-50)	ns
**CRS, n (%)**	6 (100%)	6 (85.71%)	ns
**Nasal polyps, n (%)**	3 (50%)	5 (71.43%)	ns
**Perennial AR, n (%)**	6 (100%)	7 (100%)	ns
**Seasonal AR, n (%)**	6 (100%)	7 (100%)	ns
**DSS nasal obstruction, Me (25-75%)**	2 (1-3)	3 (2-3)	ns
**DSS itching, Me (25-75%)**	0.5 (0-2)	2 (1-3)	ns
**DSS sneezing, Me (25-75%)**	1 (0-2)	1 (0-2)	ns
**DSS watery eyes, Me (25-75%)**	0 (0-1)	2 (0-3)	ns
**DSS score, Me (25-75%)**	1 (0.5-1.25)	1.75 (1.25-2.5)	ns
**mRQLQ score, Me (25-75%)**	3 (2-3)	3 (2-4)	ns
**GINA treatment step, Me (25-75%)**	2 (2-4)	3 (2-4)	ns
**ACQ6 score, Me (25-75%)**	1 (0-2)	1 (1-2)	ns
**Eos abs. [cells/uL], Me (25-75%)**	220 (180-230)	490 (140-650)	ns
**Asthma control (GINA)**			
**Well controlled, n (%)**	4 (66.67%)	3 (50%)	ns
**Partly controlled, n (%)**	1 (16.67%)	2 (33.33%)	ns
**Uncontrolled, n (%)**	1 (16.67%)	1 (16.67%)	ns
**Asthma severity (GINA)**			
**Mild, n (%)**	2 (33.33%)	2 (33.33%)	ns
**Moderate, n (%)**	4 (66.67%)	3 (50%)	ns
**Severe, n (%)**	0 (0%)	1 (16.67%)	ns
